# Community Resilience Learning Collaborative and Research Network (C-LEARN): Study Protocol with Participatory Planning for a Randomized, Comparative Effectiveness Trial

**DOI:** 10.3390/ijerph15081683

**Published:** 2018-08-07

**Authors:** Benjamin F. Springgate, Armen C. Arevian, Ashley Wennerstrom, Arthur J. Johnson, David P. Eisenman, Olivia K. Sugarman, Catherine G. Haywood, Edward J. Trapido, Cathy D. Sherbourne, Ashley Everett, Michael McCreary, Diana Meyers, Sheryl Kataoka, Lingqi Tang, Jennifer Sato, Kenneth B. Wells

**Affiliations:** 1 LSU Health Sciences Center, New Orleans School of Medicine, New Orleans, LA 70112, USA; okacsi@lsuhsc.edu (O.K.S.); aevere@lsuhsc.edu (A.E.); jsato@tulane.edu (J.S.); 2 LSU Health Sciences Center, New Orleans School of Public Health, New Orleans, LA 70112, USA; etrapi@lsuhsc.edu; 3 UCLA Semel Institute for Neuroscience and Human Behavior, Research Center for Health Services and Society, Los Angeles, CA 90024, USA; aarevian@mednet.ucla.edu (A.C.A.); mmccreary@mednet.ucla.edu (M.M.); skataoka@mednet.ucla.edu (S.K.); ltang@mednet.ucla.edu (L.T.); kwells@mednet.ucla.edu (K.B.W.); 4 Tulane University School of Medicine, New Orleans, LA 70112, USA; awenners@tulane.edu; 5 Center for Sustainable Engagement and Development, New Orleans, LA 70117, USA; ajohnson@sustainthenine.org; 6 David Geffen School of Medicine at UCLA and UCLA Center for Public Health and Disasters, Los Angeles, CA 90095, USA; DEisenman@mednet.ucla.edu; 7 Louisiana Community Health Outreach Network; New Orleans, LA 70119, USA; chaywoo@tulane.edu; 8 The RAND Corporation, Santa Monica, CA 90401, USA; cathyd@rand.org; 9 St. Anna’s Episcopal Church, New Orleans, LA 70116, USA; diana@stannanola.org

**Keywords:** community resilience, social determinants of health, disaster resilience, mental health

## Abstract

This manuscript presents the protocol and participatory planning process for implementing the Community Resilience Learning Collaborative and Research Network (C-LEARN) study. C-LEARN is designed to determine how to build a service program and individual client capacity to improve mental health-related quality of life among individuals at risk for depression, with exposure to social risk factors or concerns about environmental hazards in areas of Southern Louisiana at risk for events such as hurricanes and storms. The study uses a Community Partnered Participatory Research (CPPR) framework to incorporate community priorities into study design and implementation. The first phase of C-LEARN is assessment of community priorities, assets, and opportunities for building resilience through key informant interviews and community agency outreach. Findings from this phase will inform the implementation of a two-level (program-level and individual client level) randomized study in up to four South Louisiana communities. Within communities, health and social-community service programs will be randomized to Community Engagement and Planning (CEP) for multi-sector coalition support or Technical Assistance (TA) for individual program support to implement evidence-based and community-prioritized intervention toolkits, including an expanded version of depression collaborative care and resources (referrals, manuals) to address social risk factors such as financial or housing instability and for a community resilience approach to disaster preparedness and response. Within each arm, the study will randomize individual adult clients to one of two mobile applications that provide informational resources on services for depression, social risk factors, and disaster response or also provide psychoeducation on Cognitive Behavioral Therapy to enhance coping with stress and mood. Planned data collection includes baseline, six-month and brief monthly surveys for clients, and baseline and 12-month surveys for administrators and staff.

## 1. Introduction

There are substantial health impacts from environmental disasters, including exacerbation of pre-existing illnesses, risk from exposure to toxins, injury, increased risk for chronic illnesses from aggravation of structural determinants of health [1,2,3,4,5,6,7] and threats to mental health [8,9,10,11,12,13]. There is increasing attention to the effects of disasters on mental health, such as depression, anxiety and post-traumatic stress disorder [14,15,16,17,18]. There are multiple consequences of disasters that may cause distress including: (1) physical injury/trauma; (2) displacement and damage to housing and property; (3) separation from loved ones; (4) loss of employment; (5) disrupted social networks and supports; (6) services redistribution; and (7) hazard exposure [8]. In addition, the risk of exposure to these consequences is greater for under-resourced groups that are also more likely to face adverse social determinants of health and less access to services. Groups that are particularly vulnerable to adverse consequences include children, the elderly, lower-income individuals, and more geographically isolated communities [1,19,20,21].

There is increasing focus on individual and community-level resilience to reduce the impact of recurrent environmental disasters including in regions affected by climate change [22,23]. Resilience has been variously defined, including as “a dynamic process encompassing positive adaptation within the context of significant adversity” and incorporating recovery, or return to satisfactory quality of life [1]. The United Nations has defined resilience as “the ability of a system, community or society exposed to hazards to resist, absorb, accommodate, adapt to, transform and recover from the effects of a hazard in a timely and efficient manner, including through the preservation and restoration of its essential basic structure and functions through risk management [24]”. Similarly, community resilience refers to capacities of communities to withstand challenges such as disasters and promote resilience across individuals [22,25]. Domains of community resilience include social inclusion and connectedness, stakeholder participation in planning and development of valued resources, and built environments encouraging collaboration [26]. Enhancing community resilience is a national goal in the United States, with recommendations to integrate efforts across sectors [27]. Such efforts may also improve individual resilience by increasing social networks and coping resources [23,28]. Interventions to build community resilience may also promote social capital, assets, and service sector collaboration [26,27,28,29,30]. In the context of disasters, events may have direct effects on mental health or indirect effects through physical health, social, or environmental consequences. For example, a longitudinal study of women near the Gulf Oil Spill of 2010, found increases in depression, mental distress and domestic violence associated with physical exposure and economic distress [11,12,13]. However, providing coping resources, social supports and services can be protective against such negative impacts [8,31,32]. It is also important to note that resilience may also differ by gender, age, socioeconomic status, ethnicity and other factors [33,34,35,36,37].

Social determinants of health, including community contextual factors (e.g., poor housing or societal barriers to inclusion) and individual-level exposure (e.g., homelessness) contribute to physical and mental health disparities, with low socioeconomic status as an overarching causal factor [38,39,40,41,42,43,44,45] and as noted above, disasters may exacerbate prevalence and impact of adverse social determinants. The literature cited above on resilience in the context of social determinants suggests employing a socio-ecological framework to promote resilience to multiple types of adversity, as disasters and their impacts occur in the context of pre-existing individual, social, and environmental factors [46]. Within such a framework, maintaining mental health-related quality of life by addressing depressive symptoms that compromise diverse areas of functioning may facilitate individual and community capacities to activate protective factors—consistent with the Sendai Framework for Disaster Risk Reduction, an international framework that recognizes the importance of an integrated, public health approach to disaster resilience that includes a response to mental health impacts [47]. Further, efforts to foster resilience may benefit from building specific capacities at community and individual levels to address multiple and at times potentially inter-related sources of adversity (i.e., depression, social determinants, disaster exposure).

Determining how to best achieve the balance of community and individual capacities to support resilience may be key to resilience practice and policy areas threatened by climate change and environmental disaster risk. However, there are few studies that compare the effectiveness of approaches to improve mental health-related quality of life through addressing resilience related to depression, social determinants of health, and disaster response. To help fill that gap, the National Academy of Sciences’ Gulf Research Program funded the “Community Learning and Resilience Research Network” (C-LEARN) in Southern Louisiana, as the founding sponsor [48]. C-LEARN uses a Community Partnered Participatory Research (CPPR) approach to address depression, disaster recovery, and preparedness [49,50]. CPPR supports academic and community partners in equitable collaboration through principles of power sharing, trust and respect, and two-way knowledge exchange in program development, implementation, and experimental research. Initial applications of this approach to engage under-resourced communities in South Los Angeles in addressing depression were blended with evidence-based depression collaborative care for healthcare settings to support mental health recovery post-Katrina in New Orleans through the Mental Health Infrastructure and Training (MHIT) project [23,51,52,53,54,55,56,57,58].

In addition, a multi-sector model for community disaster resilience was compared to standard disaster preparedness coalitions in the Los Angeles County Disaster Resilience (LACCDR) initiative [23,28,59,60]. LACCDR developed innovative approaches to measure community resilience, including tabletop exercises and network surveys [23,60].

This manuscript describes the planned design, aims, and methods for the C-LEARN study, a two-level (program and individual) randomized trial of alternative approaches to promote resilience in Southern Louisiana. The design was developed in full partnership with community stakeholders.

## 2. Study Design

### 2.1. Aims

C-LEARN has the overall aim of determining how best to improve resilience, particularly mental health-related quality of life for individual adult clients of diverse health and community-social service programs, through alternative strategies to build capacity of programs to provide services for depression, social risk factors and disaster-related concerns, as well as through alternative forms of individual client information technology support for addressing the same range of issues.

Specific aims are:

1. To engage communities in South Louisiana in a community learning initiative on how to best build capacity to enhance resilience to depression, adverse social determinants of health, and disaster exposure. This aim includes a qualitative assessment of local community resilience priorities and assets to inform study implementation.
2. To compare the effectiveness for improving mental health quality of life (MHRQL) (primary) and coping with stressors and other resilience outcomes (secondary), of two program-level interventions to build capacity for resilience programs: (1) Technical Assistance (TA) to individual programs vs. (2) Community Engagement and Planning (CEP) to support multi-sector coalitions. Hypothesis: CEP will be more effective at enhancing individual client (primary and secondary) outcomes. In addition, CEP will be more effective than TA in engaging programs and providers in trainings to improve services for depression, social risks and disaster concerns (primary), and in increasing the use of such services by programs and providers (secondary).
3. To compare the effectiveness for improving MHRQL and other resilience outcomes of two mobile apps: CR and CR+eCBT: (1) CR—An app providing only information on community resources, or (2) CR+eCBT—An app providing information on community resources and education on a cognitive behavioral therapy (eCBT) based approach to enhance individual resilience (i.e., coping with mood and stressors). Hypothesis: CR + eCBT mobile app will be more effective CR in improving the same primary and secondary client outcomes as for Aim 2. To describe strategies from CEP coalitions used to address depression, social determinants and disaster resilience, to inform interpretation and dissemination of findings.

### 2.2. Design

**Figure 1:**
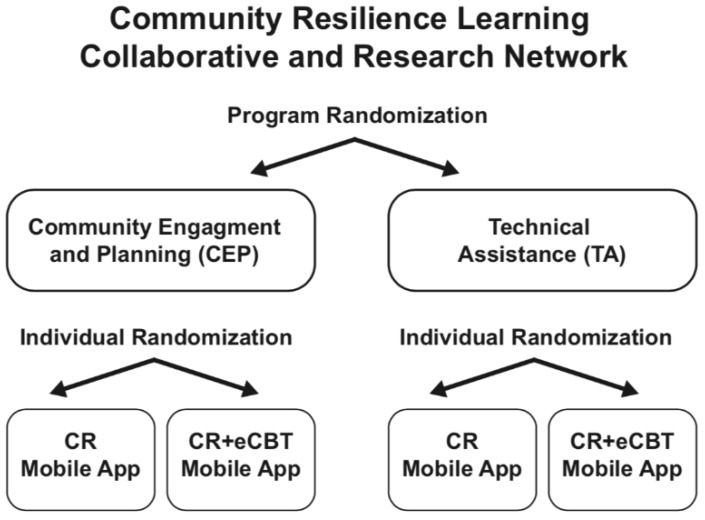
Community Resilience Learning Collaborative and Research Network Study Design.

As shown in Figure 1, the design has an overall Community Partnered Participatory Research (CPPR) approach to implement a 2 by 2, randomized comparative effectiveness trial. Randomization occurs at the program level to either CEP or TA, where a program is a discrete services program with its own staff and clients; there may be multiple programs within a given administrative agency, including different geographic sites such as clinics. Further, programs may offer services in different content areas, such as physical health, mental health, social services, disaster services, faith-based, etc., referred to as different “sectors”. In addition, individual participants will be randomized to one of two mobile apps for coping with stressors and disasters.

The project and design phase has been led by a Leadership Council, including academic, community, and health system participants who have guided all aspects of the study, and operate under CPPR principles [49]. Initial leaders are academic and community partners from the Community and Patient Partnered Research Network (CPPRN) across South Louisiana and Los Angeles with additional stakeholder advisors from New Orleans, Baton Rouge and Coastal South Louisiana planned for as engagement of communities proceeds [61]. Through the assessment of stakeholder priorities (Aim 1), potential partners have been identified from different communities. The Council has an Executive Committee and committees on Interdisciplinary Methods (design, measures, analysis), Interventions, and Operations, with academic and community stakeholder leadership in each. As a CPPR initiative, the design has been refined with stakeholder input under a pre-specified, participatory process with initial design elements finalized prior to program recruitment and final design elements to be completed prior to client recruitment. The Council developed a Memorandum of Understanding that summarizes collaboration principles, leadership responsibilities, and issues such as data access, reviewing and sharing publications, and handling conflict. The Executive Committee meets weekly, Council biweekly to monthly, and work groups are meeting weekly or biweekly. The Council reviews work group recommendations and facilitates larger community input and approval through a stakeholder advisory committee and larger community conferences, one of which occurred prior to publishing this phase (pre-program recruitment) of the protocol.

### 2.3. Interventions

The main comparators are CEP and TA. Healthcare and community-based programs that are assigned to CEP and TA will both receive training and support for implementation of an expanded model of evidence-based depression collaborative care that also addresses social determinants and disaster readiness. The depression toolkits to be used are from studies on adults, including, racial/ethnic minority and low-income groups, with community health worker manuals from prior work in New Orleans, adapted for community-based programs in the Community Partners In Care (CPIC) study [51,52,53,55,62]. Toolkits use a team-based, stepped-care approach supporting assessment, referral and treatment, outcomes monitoring and care adjustment with specialty supervision and case managers for coordination and client education. While based on components of collaborative care for depression (clinical assessment and medication management for physicians; clinical assessment and CBT for licensed counselors; case management support for screening, education and patient activation, problem solving, care coordination and outreach; team management support), the interventions will also include resources to address main social determinants (e.g., poverty/financial planning, housing resources) and disaster preparedness/response, such as online resources developed after LACCDR [60]. Initial adaptations have been made with stakeholder input, but work groups will continue to refine some materials prior to client recruitment. The differences between CEP and TA are described in the following sections.

#### 2.3.1. CEP for Coalitions

CEP creates multi-sector networks to collaborate in evidence-based and community-prioritized toolkits or intervention materials [59,63]. CEP supports a series of biweekly to monthly meetings to develop network and individual program capacity, prepare stakeholders as co-leads, and create a written training plan following CPPR principles [49,64]. CEP councils consider local context, i.e., cultural assets and stakeholder input. Disaster preparedness and public health sectors will be encouraged to offer education/resources on social determinants and disasters within CEP training plans. CEP will be supported by a Learning Collaborative, meeting 2–3 times, using activities such as project examples, tabletop exercises and self-assessment to identify gaps and formulate goals for improvement [60,64].

#### 2.3.2. TA for Individual Programs

TA uses experts to train program staff via webinars and site visits, using the same toolkits as CEP, in a “train the trainer” approach with outside referral for intensive support such as for full supervision in CBT. TA provides a series of webinars and as needed primary care site visits, focused on team support for assessment, treatment support as appropriate with medication and/or psychotherapy, case management and educational resources and access to intervention toolkits. TA experts may include a psychiatrist, CBT expert therapist, case manager, support staff and community leader to engage service programs. The team will include experts on components such as financial planning and disaster preparedness.

#### 2.3.3. Individual-Level Mobile Apps

We compare two mobile apps created as part of this study (referred to as CR and CR + eCBT) that permit interactive text messaging, mobile web, or interactive voice response (IVR) interactions, using an information technology platform (Chorus) specifically designed for participatory development [61]. Each mobile app will be adapted through workgroups with stakeholders in order to tailor content to each community. The CR app will primarily provide informational resources and referral information relevant to the local community. We will identify resources for depression, social determinants and disaster preparedness and response within each community during planning with local stakeholders. The CR + eCBT app consists of the functionality of the CR app along with an interactive component to support CBT-informed coping with mood and stressors at the individual level. This component was developed previously by our group using participatory methods with community partners and includes interactive support to enhance social support networks, support cognitive restructuring (framed through partnered input as “Catch it, Check it, Change it”), and encourage pleasant activities [61]. Participants will receive text message notifications (with frequency set by participants, up to several times per day) and can either reply back to messages to explore content or click a link in the message to access the interactive mobile app.

#### 2.3.4. Driver Diagram

**Figure 2:**
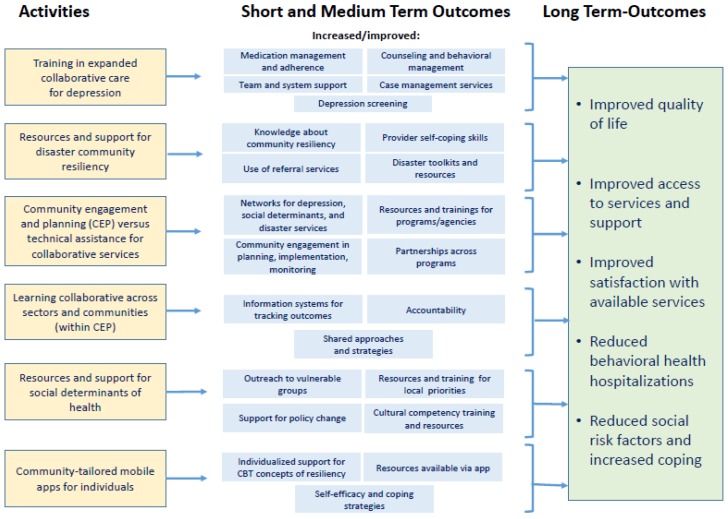
C-LEARN Logic Model.

Building on the literature above and our prior work and stakeholder input, we formulated a logic model (Figure 2), specifying expected outcomes, main drivers and intermediate processes, and key intervention features. This logic model informs the measurement framework.

### 2.4. Measures

#### 2.4.1. Measures for Client/Community Participants

We consider MHRQL (MCS-12) as the primary outcome. We include as secondary outcomes depression (PHQ-8) and physical health quality of life (PCS-12), mental wellness, homelessness risk factors and behavioral health hospitalization as community-prioritized; and a measure of general resilience. Mediating factors are self-efficacy in coping with depression, social risk factors and disaster threats, social contacts and use of health and social-community and disaster services for these concerns [32,65]. We will assess sociodemographic factors (age, gender, race/ethnicity, education, insurance coverage, family composition, family income, employment status) as moderators. We also assess exposure to social risk factors, disasters exposure and concern, post-traumatic stress disorder symptoms, presence of chronic medical conditions, and perceived community efficacy in dealing with disasters [66,67]. We also have the option of tailoring follow-up measures to specific events (e.g., floods, oil spill) [11,68]. We will explore utilizing open-ended answers to “how are things going now?” to identify linguistic markers of resilience (e.g., emotional positivity) [69]. Measures have been formulated with stakeholder leader input.

#### 2.4.2. Administrator/Provider Measures

Administrator measures for baseline and 12 months include organization partnerships for depression, social risk factors and disaster services. We will also assess program size, services offered, aspects of organizational structure, and at follow-up, participation in planning and trainings [60,62,70]. Provider or staff measures include time spent providing community services and intervention-relevant practices (e.g., problem-solving, screenings for depression, social risk factors, and disaster preparedness) (primary), and self and community efficacy to address stressors and disasters, and at baseline, training and job status [60,63,71]. These measures have received Council and community stakeholder input.

### 2.5. Randomization

#### 2.5.1. Program-Level (CEP vs. TA)

Within each community, we will randomize programs or program clusters stratified by type of content area or sector (healthcare, social-community, disaster preparedness) for program services and major geographic area, randomizing within matched program pairs to CEP or TA [62,72]. All interventions are “encouragement” interventions. That is, participants are encouraged to consider interventions to which they are assigned, no one is forced to follow a given protocol. This is more of a public health rather than a clinical approach to intervention implementation.

#### 2.5.2. Individual-Level

Clients will be randomized to receive one of the two mobile apps (CR or CR + eCBT), at the time of enrollment, with the assigned model delivered through the app.

### 2.6. Sampling

#### 2.6.1. Communities

With stakeholder input, we will recruit services programs from multiple communities in New Orleans and Coastal South Louisiana, (1–2 communities per area or up to four total). Racial and ethnic compositions of Louisiana communities are primarily African-American and Caucasian, with smaller populations of Latinos and also Vietnamese-Americans in coastal areas. The stakeholder Council has recommended specific communities with history of disaster exposure and diverse populations.

#### 2.6.2. Programs

As in CPIC, we will select eligible programs by combining program lists with community nominations, classifying programs as mental health, substance abuse, primary care/FQHCs/public health (health) or social services, community centers, parks and recreation community centers, businesses, salons, gyms, faith-based, and disaster preparedness/response programs (community) [73]. We will invite programs through letters, phone calls and visits to recruit at least 10 per community, expecting at least 60 total programs.

#### 2.6.3. Administrators and Providers/Staff

Each program will be asked to identify an administrator (N = 60 or more) that we invite to enroll and complete web-based surveys describing programs and partners at baseline and 12-month follow-up. Administrators will identify potentially eligible staff (full time, licensed or nonlicensed, but having direct client/community member contact) that we invite to enroll through meetings and notices, anticipating 2–3 service providers or staff members per program to consent for surveys (N = 120 or more). All eligible staff may participate in trainings, tracked by sign-in logs, whether or not they participate in the survey subsamples.

#### 2.6.4. Clients/Community Members

Within enrolled programs, we will ask adults aged 18 or older seeking services or at events such as health fairs, and adults accompanying minors to complete self-or interviewer-administered eligibility screeners. Individuals will be eligible if they screen positive in the last six months for exposure to social-risk factors (e.g., homelessness, below federal poverty level), disaster exposure or concern about future disasters, or using disaster services; or depression by eight-item Patient Health Questionnaire (PHQ-8) score great than or equal to 10. We expect about 70–90% to be eligible with 25–30% depressed. We will exclude persons who: (1) do not provide contact information; (2) are severely cognitively impaired by survey-staff judgment; (3) are non-English speakers; (4) do not expect to live in South Louisiana over the following 12 months. We expect to screen at least 20 individuals per program to enroll 1200–1440 participants.

#### 2.6.5. Data Collection

We will ask participants to complete baseline and six-month surveys online or by telephone interview. In addition, we will make available brief (10 min) surveys monthly via web, text-message or interactive voice on the primary and a few secondary outcomes.

#### 2.6.6. Human Subjects Protection

All procedures will have prior review and approval from the LSU Health Sciences Center-New Orleans (LSUHSC-NO) Institutional Review Board (IRB), and partnering research institutions will enter into reliance agreements with LSUHSC-NO. The study currently has IRB approval for design planning, stakeholder interviews, conferences and for development with stakeholder input and notetaking for design materials, measures and other study materials as well as for specific community engagement events such as a kick-off conference with stakeholder input. In sequence, amendments will address program, administrator, provider and recruitment and participation in surveys, intervention, and other activities.

#### 2.6.7. Power Calculations

The power calculations were based on expecting a percentage point difference between groups ranging from 8 to 10. With enrolling 720 or 600 individuals per group (1440 or 1200 in total), 80% power was projected to detect the difference between groups ranging from 9.3% to 10.7% with alpha level of 0.05 (two-sided) and ICC = 0.01, allowed a retention rate of 65–75% at six months follow-up [72]. For outcome measures assessed monthly during the six-month period, the proposed sample size with 60% retention is adequate for a between group difference of 7.3–7.9%.

### 2.7. Proposed Analysis

#### 2.7.1. General Issues

(a) Modeling. We will use an intent-to-treat (ITT) framework and hierarchical approach with information on programs, service providers/staff, and clients. We will conduct bivariate analyses to identify potential covariates for multiple regressions and compare unadjusted and adjusted intervention coefficients to assess confounding. We will explore transformations and two-part models for skewed counts with smearing estimates for retransformation; (b) Missing data. For missing data (item or survey non-response), we will use logical imputation for items as appropriate and hot-deck multiple imputation using a predictive mean-matching method for item non-response [74,75]; (c) Weights. We will create enrollment weights to represent intended populations [76,77]; (d) Multiple Comparisons. We will consider methods that incorporate bounds on probability of false findings of significance, e.g., false discovery rate [78]; (e) Multilevel Data: We will apply multi-level (i.e., hierarchical or random coefficient) models to account for clients within programs and three-levels for longitudinal data having repeated measures within clients [72].

#### 2.7.2. Aim 2 Analysis (CEP vs. TA)

The main analysis is of client outcomes. Using client/community member baseline and six-month follow-up, we will evaluate CEP versus TA effects on primary (MHRQL) and secondary/exploratory outcomes (e.g., depression, mental wellness, homelessness risk factors, behavioral health hospitalizations; physical activity, productivity, self and community efficacy for coping, use of service for depression, social determinants and disaster preparedness and response). For secondary analysis of programs, outcomes are training participation and partnerships with other service programs for depression, social determinants and disaster threat/exposure, examining intervention effects controlling for type of service sector for programs, and for community, reporting chi-square statistics. For staff/providers, also considered secondary, main outcomes are participation in training, providing community services and using problem-solving and other strategies for depression, social risk factors and disaster threat/exposure based on data from 12-month follow-up and logs at training events. In two-level hierarchical models, we will compare intervention effects on hours in training using two-part models for skewed distributions [63,79].

#### 2.7.3. Aim 3 Analysis: (CR App vs. CR + eCBT App)

In regression models above (six-month endpoint or longitudinal), we will include as intervention indicators, CEP vs. TA, CR app vs. CR + eCBT app, and their interaction. From estimated models, we will contrast a linear combination of coefficients to estimate CR vs. CR + eCBT effects within each CEP or TA group and average effects across these interventions; and conduct stratified analyses by client baseline measures.

#### 2.7.4. Qualitative Analysis

We will use several qualitative data collection methods [80]. We will use recorded interviews to assess stakeholder priorities (Aim 1), coalition strategies and strengths, and trends in services and disaster response (Aim 4). We will conduct case studies of CEP coalitions to describe coalition development, toolkit modification and training implementation using meeting notes, written plans, interviews, and group discussions at learning collaboratives [81,82].

#### 2.7.5. Linguistic Predictors

We will explore the feasibility of identifying linguistic predictors of resiliency to inform future research. For this purpose, we will use longitudinally collected, open-ended responses on the monthly brief surveys and use automated text transcription algorithms to extract lexical and vocal acoustic features, associated with hope and stressor response [69,82,83,84,85].We will use supervised learning models (i.e., support vector machine) to explore linguistic features as resilience predictors, i.e., mental wellness and MHRQL.

#### 2.7.6. Mixed-Methods Analyses

For thematic analyses we will enter data into MAXQDA software (version 12, VERBI GmbH, Berlin, Germany). Academic and community members identify themes coding 5–10 percent together and resolving discrepancies by consensus [86,87,88,89]. We will code data deductively based on study goals, e.g., training plans, and inductively based on emergent themes, e.g., new priorities [90]. We will conduct comparative analyses to identify themes across coalitions and triangulate results to describe context, strategies to address resilience, implementation strategies and outcomes. All analyses will be partnered with stakeholders [91].

#### 2.7.7. Partnered Synthesis

We will use all sources of data in community conferences to support academic and community partners in generating research, community improvement and policy recommendations, following the model of CPPR that we have applied in Louisiana and Los Angeles [54,62,92].

## 3. Discussion

We have described the study protocol for C-LEARN, a unique study with potential if successfully implemented to inform practice and policy while enhancing knowledge in the intersecting areas of community and individual resilience, disaster risk, mental health, and social risk factors for health. C-LEARN is designed to utilize a resilience-focused CPPR engagement and partnered research process across all phases of study design, implementation, findings and dissemination. This goal of enhancing public participation in research is consistent with the study’s main focus on community resilience. This phase represents the main design plans in response to Council and initial stakeholder input. The study is designed specifically to inform the added value of a multi-sector coalition approach across diverse programs versus an individual program technical support model, to build capacity for improving services for depression, social risk factors and disaster concerns—the main focus of program-level randomized design. In addition, the study is designed to inform the added benefit for individual clients of mobile health technologies that provide information on local services resources or that in addition provide interactive education on a CBT-based approach to support coping with diverse stresses, including mood symptoms, social risk factors and disaster concerns.

The implementation of the interventions and changes in services processes to improve outcomes at client, program and provider/staff over the span of three years requires attention to practicality, necessitating and building on local stakeholder priorities for important processes in local context. This study context may include pressing considerations in addressing disaster risk in a timely manner, such as promoting resilience with limited resources in a period of accelerating disaster risk and climate change. At the same time, this very context highlights the value of information gained on intervention strategies that may contribute to community resilience (program partnerships and capacities) and individual resilience, defined here as mental health-related quality of life and coping with diverse stressors, and other resilience-related outcomes. Even modest effects of interventions may hold promise for exploring new directions for practice and policy.

To date, stakeholder leaders have both co-developed the design and affirmed its relevance to local communities, further reinforced in larger stakeholder meetings, including the recent main “kick-off” conference event. However, inherently the CPPR process used in the study is designed to adapt designs to cultural priorities and assets in local areas, which may inform processes of specific intervention conditions as they are finalized and implemented, or may have implications for features such as additional community-prioritized, secondary outcomes. For example, the design was reviewed by 37 stakeholders at a kick-off event in New Orleans on 22 June 2018. Examples of issues raised were the importance of inclusion of under-resourced groups at risk for disparities in terms of disaster exposure and access to services; the importance of attending to both histories of disaster exposure and individual variation in exposure and response and ongoing threats and concerns while monitoring well-being; the importance of cultural competence in services; the need to attend to underlying social determinants; and including education for the most vulnerable including those with mental illness on issues such as access in disasters to medications. Overall, strong support for the proposed design was voiced by consumer, provider, community member and policy stakeholders. If further design changes occur, they will be included in an update prior to client recruitment and publication of main results. The planned design and stakeholder process builds on more than a decade of experience in applying CPPR to research and intervention design and implementation related to resilience and MHRQL in New Orleans and Los Angeles [54,55,62,92].

Limitations of the study include the potential for contamination with randomization of programs within the same communities in South Louisiana. We will strive to limit contamination and attempt to track contamination risks. In addition, as designed, the study is an ambitious study in terms of implementation and system change within a limited time period, which could lead to small effect sizes. However, we build on the experience of a similar comparison of CEP and TA in Los Angeles; pilot work on the App interventions, and partnership development in Southern Louisiana (increasing feasibility) [54,61,62,63,71]. The main limitations also represent a conservative bias, in that they would tend to reduce observed differences in the main intervention comparisons.

C-LEARN is likely to generate a range of additional questions and innovations worth exploring in subsequent work. For example, we recognize that it will be important to explore how to expand the broadened collaborative care model from its historic focus on depression and anxiety care to additionally address social determinants of health, building on the formative process in MHIT and CPIC [62,92]. We anticipate that innovations will emerge from the work of the coalition arms, and for this reason have included a specific aim of describing the process and products of the coalitions in that arm. This will likely inform both interpretation of observed effects as well as potential dissemination through qualitative examples for next research steps as well as practice in building capacity for resilience.

## 4. Conclusions

The C-LEARN study is a planned, randomized, comparative effectiveness trial including both community-level and individual-level interventions, including community-tailored mobile health technologies and implemented through a partnered, participatory process with stakeholder input in all phases. The study supports a needed integration across diverse service sectors usually studied separately: disaster resilience, mental health and social determinants of health.

## References

[1] Portier C., Thigpen T.K., Carter S., Dilworth C. (2010). A Human Health Perspective on Climate Change: A Report Outlining the Research Needs on the Human Health Effects of Climate Change.

[2] Greenough G., McGeehin M., Bernard S.M., Trtanj J., Riad J., Engelberg D. (2001). The potential impacts of climate variability and change on health impacts of extreme weather events in the United States. Environ. Health Perspect..

[3] Sullivan S.M., Peters E.S., Trapido E.J., Oral E., Scribner R.A., Rung A.L. (2016). Assessing mediation of behavioral and stress pathways in the association between neighborhood environments and obesity outcomes. Prev. Med. Rep..

[4] Woodward A.J., Samet J.M. (2018). Climate Change, Hurricanes, and Health. Am. J. Public Health.

[5] Lane K., Kizzy C.-G., Wheeler K., Abid Z., Graber N., Matte T. (2013). Health Effects of Coastal Storms and Flooding in Urban Areas: A Review and Vulnerability Assessment. J. Environ. Public Health.

[6] Ryan B., Franklin R.C., Burkle F.M., Aitken P., Smith E., Watt K., Leggat P. (2015). Identifying and Describing the Impact of Cyclone, Storm and Flood Related Disasters on Treatment Management, Care and Exacerbations of Non-communicable Diseases and the Implications for Public Health. PLOS Curr. Disasters.

[7] Barbeau D.N., Grimsley L.F., White L.E., El-Dahr J.M., Lichtveld M. (2010). Mold Exposure and Health Effects Following Hurricanes Katrina and Rita. Annu. Rev. Public Health.

[8] Stanke C., Murray V., Amlôt R., Nurse J., Williams R. (2012). The effects of flooding on mental health: Outcomes and recommendations from a review of the literature. PLoS Curr..

[9] Berry H.L., Bowen K., Kjellstrom T. (2010). Climate change and mental health: A causal pathways framework. Int. J. Public Health.

[10] Fritze J.C., Blashki G.A., Burke S., Wiseman J. (2008). Hope, despair and transformation: Climate change and the promotion of mental health and wellbeing. Int. J. Ment. Health Syst..

[11] Gaston S., Nugent N., Peters E.S., Ferguson T.F., Trapido E.J., Robinson W.T., Rung A.L. (2016). Exploring heterogeneity and correlates of depressive symptoms in the Women and Their Children’s Health (WaTCH) Study. J. Affect. Disord..

[12] Rung A.L., Gaston S., Oral E., Robinson W.T., Fontham E., Harrington D.J., Trapido E., Peters E.S. (2016). Depression, mental distress, and domestic conflict among Louisiana women exposed to the Deepwater Horizon oil spill in the WatCH study. Environ. Health Perspect..

[13] Rung A.L., Oral E., Fontham E., Harrington D.J., Trapido E.J., Peters E.S. (2015). Mental Health Impact of the Deepwater Horizon Oil Spill among Wives of Clean-up Workers. Epidemiology.

[14] Palinkas L.A., Petterson J.S., Russell J., Downs M.A. (1993). Community patterns of psychiatric disorders after the Exxon Valdez oil spill. Am. J. Psychiatry.

[15] Kessler R., Galea S., Gruber M., Sampson N., Ursano R., Wessel S. (2008). Trends in mental illness and suicidality after Hurricane. Mol. Psychol..

[16] Hirth J.M., Leyser-Whalen O., Berenson A.B. (2013). Effects of a Major U.S. Hurricane on Mental Health Disorder Symptoms Among Adolescent and Young Adult Females. J. Adolesc. Health.

[17] Cherry K.E., Galea S., Su L.J., Welsh D.A., Jazwinski S.M., Silva J.L., Erwin M.J. (2010). Cognitive and Psychosocial Consequences of Hurricanes Katrina and Rita Among Middle-Aged, Older, and Oldest-Old Adults in the Louisiana Healthy Aging Study (LHAS). J. Appl. Soc. Psychol..

[18] Schwartz R.M., Gillezeau C.N., Liu B., Lieberman-Cribbin W., Taioli E. (2017). Longitudinal Impact of Hurricane Sandy Exposure on Mental Health Symptoms. Int. J. Environ. Res. Public Health.

[19] Mearns R., Norton A. (2010). The Social Dimensions of Climate Change, Equity and Vulnerability in a Warming World.

[20] Gitay H., Bettencourt S., Kull D. (2013). Building Resilience: Integrating Climate and Disaster Risk into Development.

[21] Peres L.C., Trapido E., Rung A.L., Harrington D.J., Oral E., Fang Z., Fontham E., Peters E.S. (2016). The Deepwater Horizon Oil Spill and physical health among adult women in southern Louisiana: The Women and Their Children’s Health (WaTCH) study. Environ. Health Perspect..

[22] Morton M.J., Lurie N. (2013). Community resilience and public health practice. Am. J. Public Health.

[23] Chandra A., Williams M., Plough A., Stayton A., Wells K.B., Horta M., Tang J. (2013). Getting actionable about community resilience: The Los Angeles county community disaster resilience project. Am. J. Public Health.

[24] United Nations Office for Disaster Risk Reduction Terminology. https://www.unisdr.org/we/inform/terminology#letter-r.

[25] Norris F.H., Stevens S.P., Pfefferbaum B., Wyche K.F., Pfefferbaum R.L. (2008). Community resilience as a metaphor, theory, set of capacities, and strategy for disaster readiness. Am. J. Community Psychol..

[26] Aldrich D.P., Meyer M.A. (2014). Social capital and community resilience. Am. Behav. Sci..

[27] FEMA (2011). A Whole Community Approach to Emergency Management: Principles, Themes, and Pathways for Action.

[28] Plough A., Fielding J.E., Chandra A., Williams M., Eisenman D., Wells K.B., Law G.Y., Fogleman S., Magaña A. (2013). Building community disaster resilience: Perspectives from a large urban county department of public health. Am. J. Public Health.

[29] Delgado M., Humm-Delgado D. (2013). Asset Assessments and Community Social Work Practice.

[30] Ryan B., Franklin R.C., Burkle F.M. Jr., Aitken P., Smith E., Watt K., Leggat P., Luthar S., Cicchetti D., Becker B. (2015). Identifying and Describing the Impact of Cyclone, Storm and Flood Related Disasters on Treatment Management, Care and Exacerbations of Non-communicable Diseases and the Implications for Public Health. PLOS Curr. Disasters.

[31] Norris F.H., Baker C.K., Murphy A.D., Kaniasty K. (2005). Social support mobilization and deterioration after Mexico’s 1999 flood: Effects of context, gender, and time. Am. J. Community Psychol..

[32] Sherbourne C.D., Edelen M.O., Zhou A., Bird C., Duan N., Wells K.B. (2008). How a Therapy-Based Quality Improvement Intervention for Depression Affected Life Events and Psychological Well-Being over Time. Med. Care.

[33] Neria Y., Nandi A., Galea S. (2008). Post-traumatic stress disorder following disasters: A systematic review. Psychol. Med..

[34] Norris F.H. (1992). Epidemiology of trauma: Frequency and impact of different potentially traumatic events on different demographic groups. J. Consult. Clin. Psychol..

[35] North C.S., Pfefferbaum B. (2013). Mental health response to community disasters: A systematic review. JAMA J. Am. Med. Assoc..

[36] Palinkas L., Russell J., Downs M., Petterson J. (1992). Ethnic differences in stress, coping, and depressive symptoms after the Exxon Valdez oil spill. J. Nerv. Ment. Dis..

[37] Palinkas L.A., Petterson J.S., Russell J.C., Downs M.A. (2004). Ethnic differences in symptoms of post-traumatic stress after the Exxon Valdez oil spill. Prehosp. Disaster Med..

[38] Marmot M., Wilkinson R. (2006). Social Determinants of Health.

[39] Galea S., Vlahov D. (2002). Social determinants and the health of drug users: Socioeconomic status, homelessness, and incarceration. Public Health Rep..

[40] Wilkinson R.G., Pickett K.E. (2006). Income inequality and population health: A review and explanation of the evidence. Soc. Sci. Med..

[41] Jane-Llopis E., Anderson P. (2006). A policy framework for the promotion of mental health and the prevention of mental disorders. Mental Health Policy and Practice across Europe.

[42] Galea S., Ahern J., Nandi A., Tracy M., Beard J., Vlahov D. (2007). Urban Neighborhood Poverty and the Incidence of Depression in a Population-Based Cohort Study. Ann. Epidemiol..

[43] Lustman P.J., Anderson R.J., Freedland K.E., De Groot M., Carney R.M., Clouse R.E. (2000). Depression and poor glycemic control: A meta-analytic review of the literature. Diabetes Care.

[44] Wadsworth M.E., Achenbach T.M. (2005). Explaining the link between low socioeconomic status and psychopathology: Testing two mechanisms of the social causation hypothesis. J. Consult. Clin. Psychol..

[45] World Health Organization (2008). Closing the Gap in a Generation: Health Equity through Action on the Social Determinants of Health.

[46] Adger W.N., Hughes T.P., Folke C., Carpenter S.R., Rockstrom J. (2005). Social-ecological resilience to coastal disasters. Science.

[47] Aitsi-Selmi A., Egawa S., Sasaki H., Wannous C., Murray V. (2015). The Sendai Framework for Disaster Risk Reduction: Renewing the Global Commitment to People’s Resilience, Health, and Well-being. Int. J. Disaster Risk Sci..

[48] Sciences, N. A. of Gulf Research Program. http://www.nationalacademies.org/gulf/about/index.html.

[49] Jones L., Wells K. (2007). Strategies for Academic and Clinician Engagement in Community-Participatory Partnered Research. JAMA.

[50] Wells K., Jones L. (2009). “Research” in community-partnered, participatory research. JAMA - J. Am. Med. Assoc..

[51] Unützer J., Katon W., Callahan C.M., Williams J.W., Hunkeler E., Harpole L., Hoffing M., Della Penna R.D., Noël P.H., Lin E.H. (2002). Collaborative care management of late-life depression in the primary care setting: A randomized controlled trial. JAMA.

[52] Wells K.B., Sherbourne C., Schoenbaum M., Duan N., Meredith L., Unützer J., Miranda J., Carney M.F., Rubenstein L.V. (2000). Impact of Disseminating Quality Improvement Programs for Depression in Managed Primary Care. JAMA.

[53] Miranda J., Chung J.Y., Green B.L., Krupnick J., Siddique J., Revicki D.A., Belin T. (2003). Treating Depression in Predominantly Low-Income Young Minority Women: A Randomized Controlled Trial. JAMA J. Am. Med. Assoc..

[54] Springgate B.F., Wennerstrom A., Meyers D., Allen I.I.I.C.E., Vannoy S.D., Bentham W., Wells K.B. (2011). Building community resilience through mental health infrastructure and training in post-Katrina New Orleans. Ethn. Dis..

[55] Wennerstrom A., Vannoy S.D., Allen C.E., Meyers D., O’Toole E., Wells K.B., Springgate B.F. (2011). Community-based participatory development of a community health worker mental health outreach role to extend collaborative care in post-Katrina New Orleans. Ethn. Dis..

[56] Meyers D., Allien C.E., Dunn D., Wennerstrom A., Springgate B.F. (2011). Community perspectives on post-Katrina mental health recovery in New Orleans. Ethn. Dis..

[57] Ngo V.K., Centanni A., Wong E., Wennerstrom A., Miranda J. (2011). Building capacity for cognitive behavioral therapy delivery for depression in disaster-impacted contexts. Ethn. Dis..

[58] Bentham W., Vannoy S.D., Badger K., Wennerstrom A., Springgate B.F. (2011). Opportunities and challenges of implementing collaborative mental health care in post-Katrina New Orleans. Ethn. Dis..

[59] Wells K.B., Springgate B.F., Lizaola E., Jones F., Plough A. (2013). Community Engagement in Disaster Preparedness and Recovery: A Tale of Two Cities—Los Angeles and New Orleans. Psychiatr. Clin. N. Am..

[60] Eisenman D., Chandra A., Fogleman S., Magana A., Hendricks A., Wells K., Williams M., Tang J., Plough A. (2014). The Los Angeles county community disaster resilience project—A Community-Level, public health initiative to build community disaster resilience. Int. J. Environ. Res. Public Health.

[61] Arevian A., O’hora J., Jones F., Mango J., Jones L., P W., Booker-Vaughns J., Pulido E., Banner D., Wells K. Participatory Technology Development to Enhance Community Resilience. Ethn. Dis..

[62] Wells K.B., Jones L., Chung B., Dixon E.L., Tang L., Gilmore J., Sherbourne C., Ngo V.K., Ong M.K., Stockdale S. (2013). Community-partnered cluster-randomized comparative effectiveness trial of community engagement and planning or resources for services to address depression disparities. J. Gen. Intern. Med..

[63] Chung B., Ngo V.K., Ong M.K., Pulido E., Jones F., Gilmore J., Stoker-Mtume N., Johnson M., Tang L., Wells K.B. (2015). Participation in Training for Depression Care Quality Improvement: A Randomized Trial of Community Engagement or Technical Support. Psychiatr. Serv..

[64] Nembhard I.M. (2012). All teach, all learn, all improve?. Health Care Manag. Rev..

[65] Sherbourne C.D., Stewart A.L. (1991). The MOS social support survey. Soc. Sci. Med..

[66] Kroenke K., Spitzer R.L., Williams J.B. (2001). The PHQ-9: Validity of a brief depression severity measure. J. Gen. Intern. Med..

[67] Weathers F., Litz B., Herman D., Huska J., Keane T. The PTSD Checklist (PCL): Reliability, Validity, and Diagnostic Utility. Proceedings of the Annual Convention of the International Society for Traumatic Stress Studies.

[68] Chan C.S., Rhodes J.E. (2014). Measuring exposure in hurricane Katrina: A meta-analysis and an integrative data analysis. PLoS ONE.

[69] Cohn M.A., Mehl M.R., Pennebaker J.W. (2004). Linguistic markers of psychological change surrounding September 11, 2001. Psychol. Sci..

[70] Wells K.B., Tang J., Lizaola E., Jones F., Brown A., Stayton A., Williams M., Chandra A., Eisenman D., Fogleman S. (2013). Applying community engagement to disaster planning: Developing the vision and design for the Los Angeles county community disaster resilience initiative. Am. J. Public Health.

[71] Landry C.M., Jackson A.P., Tang L., Miranda J., Chung B., Jones F., Ong M.K., Wells K. (2017). The effects of collaborative care training on case managers’ perceived depression-related services delivery. Psychiatr. Serv..

[72] Murray D.M. (1998). Design and Analysis of Group-Randomized Trials.

[73] Chung B., Jones L., Dixon E.L., Miranda J., Wells K. (2010). Community Partners in Care Steering. Using a Community Partnered Participatory Research Approach to Implement a Randomized Controlled Trial: Planning Community Partners in Care. J. Health Care Poor Underserved.

[74] Little R.J.A. (1988). Missing-data adjustments in large surveys. J. Bus. Econ. Stat..

[75] Tang L., Song J., Belin T.R., Unützer J. (2005). A comparison of imputation methods in a longitudinal randomized clinical trial. Stat. Med..

[76] Groves R.M., Dillman D.A., Eltinge J.L., Little R.J.A. (2002). Survey Nonresponse.

[77] Korn E., Graubard B. (1999). Analysis of Health Surveys.

[78] Benjamini Y., Hochberg Y. (1995). Controlling the false discovery rate: A practical and powerful approach to multiple testing. J. R. Stat. Soc. Ser. B.

[79] Afifi A.A., Kotlerman J.B., Ettner S.L., Cowan M. (2007). Methods for Improving Regression Analysis for Skewed Continuous or Counted Responses. Annu. Rev. Public Health.

[80] Bernard H. (2002). Research Methods in Anthropology-Qualitative and Quantitative Approaches.

[81] Emerson R., Fretz R., Shaw L. (2011). Writing Ethnographic Fieldnotes.

[82] Mundt J.C., Vogel A.P., Feltner D.E., Lenderking W.R. (2012). Vocal acoustic biomarkers of depression severity and treatment response. Biol. Psychiatry.

[83] Hashim N.W., Wilkes M., Salomon R., Meggs J., France D.J. (2017). Evaluation of Voice Acoustics as Predictors of Clinical Depression Scores. J. Voice.

[84] Faurholt-Jepsen M., Busk J., Frost M., Vinberg M., Christensen E.M., Winther O., Bardram J.E., Kessing L.V. (2016). Voice analysis as an objective state marker in bipolar disorder. Transl. Psychiatry.

[85] Nicodemus K.K., Elvevåg B., Foltz P.W., Rosenstein M., Diaz-Asper C., Weinberger D.R. (2014). Category fluency, latent semantic analysis and schizophrenia: A candidate gene approach. Cortex.

[86] Miller W.L., Crabtree B.F. (1992). Primary care research: A multimethod typology and qualitative road map. Doing Qualitative Research.

[87] Ryan G.W., Bernard H.R. (2003). Techniques to identify themes in qualitative data. Field Methods.

[88] Saint S., Kowalski C.P., Forman J., Damschroder L., Hofer T.P., Kaufman S.R., Creswell J.W., Krein S.L. (2008). A Multicenter Qualitative Study on Preventing Hospital-Acquired Urinary Tract Infection in US Hospitals. Infect. Control Hosp. Epidemiol..

[89] MacQueen K.M., McLellan E., Kay K., Milstein B. (1998). Codebook development for team-based qualitative analysis. Field methods.

[90] Maxwell J.A. (2005). Qualitative Research Design: An Interactive Approach (2nd Edition).

[91] Creswell J. (2012). Qualitative Inquiry and Research Design: Choosing Among Five Approaches.

[92] Springgate B.F., Allen C., Jones C., Lovera S., Meyers D., Campbell L., Palinkas L.A., Wells K.B. (2009). Rapid community participatory assessment of health care in post-storm New Orleans. Am. J. Prev. Med..

